# Improve Pasture or Feed Grain? Greenhouse Gas Emissions, Profitability, and Resource Use for Nelore Beef Cattle in Brazil’s Cerrado and Amazon Biomes

**DOI:** 10.3390/ani10081386

**Published:** 2020-08-10

**Authors:** Luana Molossi, Aaron Kinyu Hoshide, Lorena Machado Pedrosa, André Soares de Oliveira, Daniel Carneiro de Abreu

**Affiliations:** 1 AgriSciences, Universidade Federal de Mato Grosso, Sinop, MT 78555-267, Brazil; luana_molossi@hotmail.com (L.M.); pedrosa@agronoma.eng.br (L.M.P.); 2 Faculty Associate, School of Economics, The University of Maine, Orono, ME 04469, USA; aaron.hoshide@maine.edu; 3 Dairy Cattle Research Lab, Universidade Federal de Mato Grosso, Sinop, MT 78555-267, Brazil; andresoli@uol.com.br

**Keywords:** agriculture, Amazon, beef cattle, Cerrado, environmental impacts, sustainable agricultural intensification, carbon footprint

## Abstract

**Simple Summary:**

Deforested areas in Brazil’s Amazon and Cerrado savannah have historically transitioned to pasture for grazing tropical beef cattle. Brazil’s projected growth in beef exports emphasizes the importance of sustainably intensifying Brazil’s cattle industry on existing agricultural land without increasing deforestation nor accelerating land conversion. We adapted a widely used simulation model for cattle, pasture, and crops to closely match two cooperating beef farms, one in the Cerrado and one in the Amazon. We then simulated the adoption of pasture fertilization, pasture re-seeding, and pasture-based grain supplementation of cattle by a model beef farm. These three sustainable agricultural intensification strategies were compared to extensive cattle grazing, the status quo in Brazil. Beef productivity and economic returns were greater for grain supplementation, followed by pasture fertilization and pasture re-seeding. Grain supplementation had the lowest greenhouse gas emissions, with less energy and nitrogen use compared to extensive grazing, as measured as a “footprint” for every unit of beef body weight produced. Pasture re-seeding and fertilization had lower greenhouse gas footprints compared to extensive; however, water and energy use and nitrogen losses were greater. Grain supplementation used more human edible livestock feed than other strategies, so pasture intensification could increase future human food availability.

**Abstract:**

Economic development, international food and feed demand, and government policies have converted Brazil’s natural ecosystems into agricultural land. The Integrated Farm System Model (IFSM) was evaluated using production, economic, and weather data collected on two cooperating farms in the Legal Amazon and Cerrado biomes in the Midwest state of Mato Grosso, Brazil. Three sustainable agricultural intensification strategies, namely grain supplementation, pasture re-seeding, and pasture fertilization were simulated in IFSM with double the beef cattle stocking density compared to extensive grazing. Livestock dry matter consumption simulated in IFSM was similar for pasture grazing estimates and actual feed consumed by beef cattle on the two collaborating farms. Grain supplementation best balanced beef production and profitability with lower carbon footprint compared to extensive grazing, followed by pasture fertilization and pasture re-seeding. However, pasture re-seeding and fertilization had greater use of water and energy and more nitrogen losses. Human edible livestock feed use was greatest for grain supplementation compared to other modeled systems. While grain supplementation appears more favorable economically and environmentally, greater use of human edible livestock feed may compete with future human food needs. Pasture intensification had greater human edible feed conversion efficiency, but its greater natural resource use may be challenging.

## 1. Introduction

Global population’s projected peak during the second half of this century is about 9.4 billion between 2060 to 2080 [1]. Food production needs to grow in a similar way to meet demand. Brazil is expected to be an important contributor to meeting global food requirements, accounting for 70% of projected production in 2050 [2]. The beef cattle industry is an important sector for the Brazilian economy, supplying animal protein for both national and international markets [3]. With a cattle herd of 221.81 million head, Brazil is the largest producer and second largest exporter of beef (~2 billion metric tons of carcass equivalent), having slaughtered more than 39.2 million cattle in 2017 [4]. The Cerrado (i.e., tropical savannah) biome is estimated to have more than 30% of Brazil’s cattle herd and is responsible for 55% of slaughtered beef in the country [5,6]. The Amazon biome makes up almost half of Brazil’s land area and supports 38% of Brazil’s cattle herd [7,8].

Economic development, international market demand for food and livestock feed, and government policies have led to the transformation of natural ecosystems into agricultural land in Brazil [9]. Agricultural expansion in Brazil’s Amazon and Cerrado biomes involving extensive grazing of beef cattle has generated effects that are not only local but global, such as increased emissions of carbon dioxide (CO_2_), due to land use conversion of forest to agricultural areas [10,11], and cattle enteric fermentation (methane or CH_4_), as well as urine and feces decomposition, which releases nitrous oxide (N_2_O) [12].

In Brazil, the agricultural sector accounts for 35% of all 2010 emissions, with more than half (56%) estimated to come from cattle (CH_4_) and an additional 18% of direct and indirect emissions of nitrous oxide (N_2_O) from animal excreta deposited on pasture mainly by cattle grazing on extensive pastures [13,14]. The recovery of degraded pastures in Brazil through sustainable agricultural intensification can reduce the need to open new agricultural frontiers through deforestation of native vegetation. 

Pastures recovered by fertilization and re-seeding can have higher yields [15], as well as lower greenhouse gas (GHG) emissions per kg of beef produced [16]. The area required to produce one kg of beef carcass in degraded pasture is approximately 320 m^2^ but falls to 45–50 m^2^ when grazing receives fertilization [14]. Similarly, grain supplementation can improve animal performance, while decreasing GHG emissions [13,17,18].

The objectives of this study were to (1) evaluate the Integrated Farm System Model (IFSM) using data collected on cooperating farms in both the Amazon and Cerrado biomes and (2) simulate the productive efficiency and environmental impacts of sustainable agricultural intensification (SAI) strategies in the Legal Amazon and in the Cerrado using IFSM. We calibrated and evaluated IFSM for these tropical climates, and this tool can assist in decision-making by farmers comparing pasture re-seeding (PS), pasture fertilization (PF), and grain supplementation (GS). These SAI strategies may have greater potential for adoption by farmers in Mato Grosso and the rest of Brazil compared to rotational grazing and integrated crop-livestock systems as they are less management intensive and are not as dependent on spatial proximity of specialized crop (e.g., soybean) and livestock (e.g., extensive beef) producers.

## 2. Materials and Methods 

### 2.1. Integrated Farm System Model

Crop-livestock simulation models reproduce the main agricultural processes to represent the production system statically over time, allowing a more complete evaluation [19]. The Integrated Farm System Model (IFSM) is a comprehensive software used to simulate milk and meat production systems for cattle [20]. Current studies of beef production compare different systems and contrast their effects on resource use and production efficiency [21], environmental impact [19,22], and production costs and net return [18]. Sub-models are included to represent all the major processes that occur on farms [23]. Thus, agricultural systems are simulated over many years of meteorological data to determine long-term performance and risk assessment from climatic variation [19].

The IFSM software is organized into six sections that predict nutrient intakes and animal performance. Series of equations are simultaneously solved to obtain an optimal solution for feeding (e.g., grain supplementation and/or pasture). Feed characteristics are defined by the user and available rations are allocated to groups of animals. The intake of nutrients is then established for all groups of animals and used to predict the body growth of animals each month of each simulated year [24]. Based on diet information, the amount and content of nutrients in bovine manure can be determined [20]. Thus, nutrient balances are determined as the sum of all imports of nutrients from feed, fertilizer, deposition and fixation of legumes, subtracting what has been withdrawn by exports of milk, feed, animal production, manure, and losses leaving the farm [25].

Environmental impacts are simulated, including emissions of gases from fertilizers, soil denitrification and leaching. Emissions of carbon dioxide and methane are modeled through their sources and sinks of crops, animals and manure to predict net emissions of greenhouse gases [26]. Greenhouse gas emissions released on-farm include those from food and animals produced and purchased, electricity, fertilizers, pesticides, and machinery [19].

The growth and development of crops and forages are predicted daily based on the availability of water and nutrients in the soil, ambient temperature and solar radiation absorbed by the system [27]. Grazing animals are kept outdoors throughout the year, but pasture growth may not be available for some periods of the year, so feeding strategy is defined by the groups of animals present in the pasture and by the amount of time they have access to the pasture. Thus, the amount of pasture consumed each month is limited by the pasture growth predicted by the model [20]. Simulation of alternative production strategies for a representative farm can identify strategies that balance livestock production and profit with lower environmental impacts [28,29].

### 2.2. Representative Farms

#### 2.2.1. Cerrado Biome Cooperating Farm

**Figure 1:**
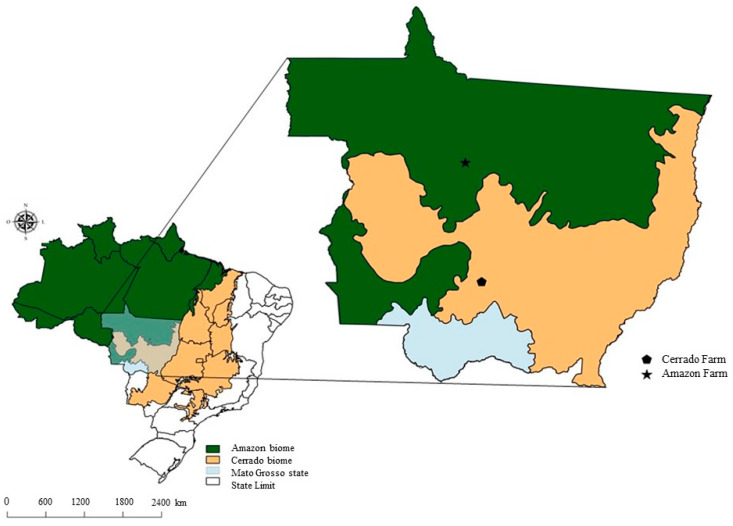
Location of cooperating farms in the Amazonia and Cerrado biome.

The cooperating beef farm in the Cerrado has 1389 ha of its own land in the central-southern region of Mato Grosso state (S 15°51′81.9″ W 56°22′26.4″) in the municipality of Acorizal (Figure 1), with 930.6 ha used exclusively for livestock and the rest of the area designated as legal forest reserve for permanent preservation of vegetation (33%). 

The cooperating farmers provided comprehensive data on its operations, including property size, equipment and facilities used, forage characteristics, number of animals, food normally purchased or produced on the farm, herd maintenance costs, inputs, and outputs (Table 1). Data were collected from June 2016 to July 2018. The climate of the region is tropical continental always warm with dry winter and rainy summer, with type “Aw” Köppen classification. The performance of the farm was simulated based on the meteorological data collected at the Rain Wise^®^ station from July 2016 to June 2017.

The farm ranches Nelore cattle (*Bos indicus*), managing breeding, stocker, and finishing cattle (Table 2) in pastures with tropical grass, predominantly *Brachiaria brizantha* cv. Marandú. The finishing phase is not carried out on-farm. When cattle body weight reaches about 360 kg, they are transferred to a confinement facility ~450 km away until they reach the ideal slaughter weight. Breeding cows and bulls receive only mineral supplementation. Finishing cattle receive multiple supplements (0.01% of body weight) specifically formulated to meet the nutritional requirements during both the wet season (October to April) and dry season (May to September).

Soil samples were collected from all pasture areas during the first half of 2016. About ten to twenty subsamples were collected from pastures. Homogenates and composite samples were sent to the laboratory of the Federal University of Mato Grosso to determine physical and chemical parameters. The cooperating farm’s soil type is deep sandy Franco. Pasture fertilization is carried out annually during the summer, according to the nutrient replenishment requirements estimated from soil analyses.

#### 2.2.2. Amazon Biome Cooperating Farm

The cooperating beef farm in the Amazon is in the municipality of Porto dos Gaúchos in the state of Mato Grosso (S 11°44′01.3″ W 56°4′55.7″). In the first year (2015–2016), farm pasture and crop area was 6147 hectares, while, in the second year, 2016–2017, 600 hectares of pasture area were added and 874 hectares were cultivated with soybeans. In the third year, the integrated area was 1214 hectares cultivated with soy.

The cooperating farm is specialized in beef cattle production, more recently diversifying management of pasture and integrating pasture areas with soy and corn. The climate is classified as tropical humid (Athi Köppen classification) with summer rainy season and winter dry season. Common soil types include the dystrophic red latosol and the quartzarenic neosol.

Full animal inventory was collected monthly and during the vaccination campaign for foot-and-mouth disease, in order to determine cattle group characteristics in addition to how the herd has evolved. The average annual beef herd was 15,769 head in 2015–2016, declining to 11,708 head over the next two years. The herd consists mainly of Nelore cattle (*Bos indicus*), with a stocking rate of 1.8 head per ha in 2017–2018. *Brachiaria brizantha* spp. and *Panicum maximum* spp. pastures are used with mineral supplementation for breeding and finishing.

Data collected from July 2015 to June 2018 included farm areas used, resource inventories, technical information on crop and pasture management, such as fertilizer application rates, dates and number of operations, labor demand, grazing period, and pasture quality. Meteorological data (solar radiation, precipitation, minimum and maximum temperature) were collected at the Rain Bird^®^ Climate Minder weather station, located on the farm (Figure 1).

### 2.3. Cooperating Beef Farms Evaluations

IFSM models were specifically calibrated to both cooperating farms for each year farm-level production data was collected for the Cerrado (2016–2017 and 2017–2018) and Amazon (2015–2016 through 2017–2018) cooperating farms. IFSM parameters were adjusted to reflect observed livestock production, purchased concentrated feeds, and/or crop yield. Pasture yield was not measured on-farm; rather, this was estimated as a range from 1.8% to 2.5% of recommended dry matter intake for each cattle group in the herd [30]. Both biomes had models set for seasonal (spring, summer, fall, and winter) variation in pasture bromatological composition [31]. Due to the complexity of the analysis to measure dry matter intake of the herd, such analyses were not performed.

### 2.4. Beef Production Systems Simulations

Once the IFSM model has been evaluated, it is possible to simulate different strategies for the sustainable agricultural intensification (SAI) of beef production system. Although the cooperating farms present productive differences, the SAI systems used in our simulations are adaptable to farms of varied scale and herd size [32]. The simulated systems were an extensive pasture system used as control, representing the most common system present in both biomes and three systems with SAI: pasture re-seeding (PS), pasture fertilization (PF), and grain supplementation (GS). 

IFSM calculates net return (profit) of the meat production system as the sum of all farm revenues minus the sum of all activity-related costs [20]. Revenues include income from animal sales, while costs include purchased feed and activities associated with animal production, such as fuel, facilities, labor, seeds, fertilizers, and chemicals. We also compared relative profitability of all systems assuming annual profits grew at a compound interest rate of 6%. Environmental impacts of beef production systems can also be simulated, such as greenhouse gas emissions and water and energy use, as well as nitrogen footprint. Since actual data on environmental impacts may not be available or quantified, such as the emission of greenhouse gases or the fixation of soil N, estimates can be obtained using models, like IFSM [22], or other computational analyses [13].

Accurate assessment and comparison of advanced systems require the use of a common size, as well as control of variation, in farm characteristics. The area used for all simulations is 1200 hectares, but the stocking rate between the extensive and sustainable agricultural intensification (SAI) systems was different, making the number of animals in the extensive system smaller due to its lower carrying capacity. The extensive stocking rate was 1 head/hectare (ha). Herd inventory was 440 cows and bulls, and 353 replacement, 179 stocker, and 229 finishing cattle. 

For SAI simulations, the stocking rate was increased to 2 head/ha, so the herd consisted of 880 cows and bulls, 705 replacement, 358 stockers, and 457 finished cattle. The number of animals was the same in all three SAI simulations to reduce undesirable variability during system comparisons. The simulated breed for all systems in both biomes was the Nelore breed predominant in tropical regions. Months until reaching ideal slaughter weight varied between systems due to differences in diets (Table 3). Calculating carbon footprint, water and energy use, and reactive nitrogen footprint per kg of edible beef produced assumed beef carcass weight is 52.2% of beef live weight for Nelore cattle [33]. Human edible feed conversion efficiency (heFCE) for both protein and energy were calculated for all simulated systems by dividing the content for these in beef by the content for these in human edible feed fed to cattle (e.g., corn grain and soybeans) using methods outlined in Ertl et al., 2015 [34]. A ratio of heFCE below one means the animal production system is less efficient corresponding to higher use of feeds that could be eaten by humans (e.g., corn grain, soybeans, etc.).

All simulated systems provided minerals for cattle and used a 108 hp (80 kW) tractor priced at US$7693. Extensive system pasture received no fertilization. Pasture stand life was 10 years before plowing (November 10) and re-seeding (PS), fertilized with 50-20-20 NPK kg/ha with lime application at 1 t/ha. PS used a medium-sized tractor with fuel consumption of 33.5 L/ha (13.4 L/h at 2.5 h/ha) based on re-seeding costs of US$169/ha [35]. Pasture utilization efficiency was set to 66% for the establishment year. Only 10% of pasture is fertilized (PF) each year at 100-50-50 NPK kg/ha and 1.5 t lime/ha. Pasture fertilization levels were surveyed from cooperating producers. For the grain supplementation (GS) simulation, cows, heifers, and stockers were fed at a supplementation level to meet 45% of recommended protein requirements [30]. Other costs and economic parameters are summarized in Supplementary Materials (Tables S1 and S2).

**Figure 2:**
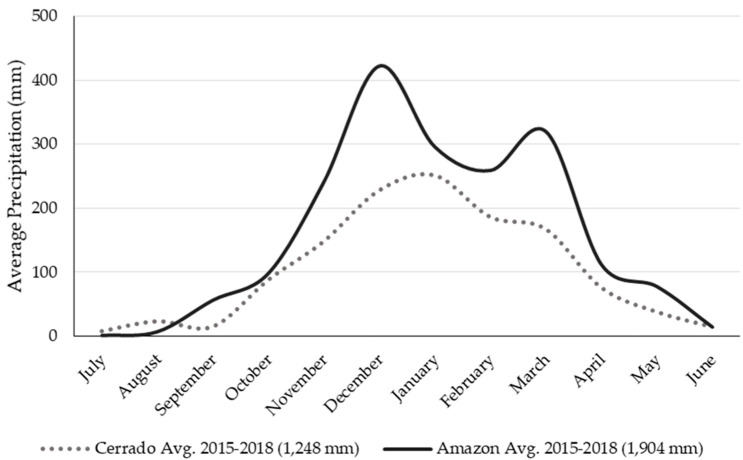
Average precipitation at cooperating farm and Cuiabá weather station in the Cerrado biome and average precipitation at cooperating farm weather station in the Amazon biome over three production years (2015–2018).

IFSM beef farm simulations for the Cerrado and Amazon biomes only differed by climatic conditions (Figure 2) and soil characteristics (Table 1). IFSM simulations are annual and not dynamically linked. Annual weather data measured from both on-farm meteorological stations in the Cerrado (2 years, 2016–2018) and Amazon (3 years, 2015–2018) biomes were supplemented with historical weather data from nearby weather stations in the Cerrado (Cuiabá, 69% of daily weather observations) and Amazon (Juara, 6% of daily weather observations) downloaded from the Instituto Nacional de Metereologia (INMET) or National Institute of Meteorology. This historical weather data (2007–2018) were used for IFSM simulations and included precipitation, solar radiation, relative humidity, and wind speed. Cuiabá was limited to 2012–2018 due to a lack of available data.

## 3. Results

### 3.1. Cooperating Farms Weather Stations

The cooperating farm in the Cerrado biome had an annual average (2016–2017 and 2017–2018) temperature of 25 °C (minimum 20 °C and maximum 30 °C) and wind speed 2.5 m/s. Solar radiation was not collected but averaged 12.4 MJ/m^2^ over these two years at the nearby Cuiabá weather station. The average precipitation for the combined observations from the cooperating farm and Cuiabá weather stations averaged 1248 mm/year from 2015–2018. For the Amazon biome cooperating farm from 2015–2016 through 2017–2018, average temperature was 28 °C (minimum 17 °C and maximum 39 °C), with averages for rainfall at 1904 mm/year, solar radiation at 17.1 MJ/m^2^, and wind speed averaged 1.2 m/s [36]. Average Amazon precipitation exceeded that in the Cerrado for most of the year (Figure 2).

### 3.2. Beef Production Systems Evalution

The simulated values for forage intake were generally lower than calculated values for the farm. Forage grasses present in tropical climate conditions (predominantly Brachiaria brizantha cv. Marandu) are adapted to the low rainfall during the dry season. IFSM optimally solved for increased supplemental feeding of forage rather than pasture grazing during this time. In reality, cattle continue to graze forage of lower nutritional quality, where its bromatological value is corrected by supplementation. Adjustments were made to narrow the gap between simulated and actual intake feeds, including reducing absorbent protein requirements by 10% [37].

#### 3.2.1. Cerrado Biome Evaluation

From 2016–2018, the cooperating farm’s beef herd and feed purchases were relatively stable. The simulated values for forage intake (t DM/ha) were between the estimated minimum and maximum values, representing 73% and 76% of the maximum value calculated for the farm for 2016–2017 and 2017–2018, respectively. For the purchase of corn grain and soybean meal (t DM/ha), the simulated value was 105% of actual for 2016–2017 and 80% of observed for the cooperating farm in 2017–2018 (Table 3). In the second year (2017–2018), cattle stocking rate increased from 1.63 to 2.52 head/ha (Table 1) due to pasture fertilization and greater grain and protein intake.

#### 3.2.2. Amazon Biome Evaluation

Simulated dry matter intake of forage by IFSM was 93%, 103%, and 76% of the values calculated for the cooperating farm across all three years (2015–2016 through 2017–2018). Simulated corn grain, cottonseed, and soybean purchases were 114%, 75%, and 100% of actual (Table 3). To meet dietary requirements of cattle, IFSM’s linear program solved for greater purchase of forage and grains instead of grazing during the dry season, due to low pasture production from lack of rain. In reality, pasture is used by cattle during the dry season. Thus, IFSM underestimated pasture dry matter intake while overestimating purchased concentrated feed.

IFSM simulated soybean yields were 109% and 103% of observed soybean yields for 2016–2017 and 2017–2018, respectively (Table 3). These small differences may be due to variations in the chemical or physical parameters of the farm’s soils. The simulated production for beef was 116% of observed production for the first year (2015–2016) since the animals spent more time to reach the ideal body weight for slaughter. In the second year (2016–2017), due to better climatic conditions and lower stocking rate associated with the integration of 13% of farm area with no-tillage, pastures improved where simulated beef production (kg BW/ha) was 97% of observed. Simulated beef production in the third year (2017–2018) was 86% of observed (Table 3). The number of cattle in the herd was higher in 2015–2016, while precipitation was lower compared to the other years. This resulted in lower forage mass production and thus higher grazing pressure. More land was leased the following year to reduce stocking density and silage was purchased for feeding.

### 3.3. Production Performance of Simulated Beef Production Systems

For Cerrado biome simulations, extensive grazed forage intake (1.61 t DM/head/year) was low due to less forage mass for cattle. Pasture re-seeding (PS) and pasture fertilization (PF) were higher at 1.71 and 1.70 DM/head/year, respectively. Both pasture intensification systems supplied nutrients to the soil, allowing greater productivity of the forage mass and, consequently, greater availability for consumption by the animals. Grain supplementation (GS) reduced forage intake to 1.56 t DM/head/year and increased feeding of concentrates (including soybean meal, 44%) three- to four-fold compared to the extensive baseline model (Table 4). Amazon biome simulations followed a similar pattern for forage intake (t DM/head/year) for extensive (1.74), PS and PF (1.77), and GS (1.63). In order to balance the nutritional requirements for the herd in IFSM’s linear programming, grain and forage purchases were made for all simulations.

Due to the better productive performance of the animals under GS, average daily gain was greater for both biomes (Table 4). Net gain of body weight at time of slaughter (kg/ha/year) of cattle in the Cerrado was greater for GS (300) than both PS and PF (281) in addition to extensive grazing (123). Amazon beef simulations had similar animal productivity (kg/ha/year) increases from extensive (124) to PS and PF (280) to GS (301). Extensive grazing’s beef productivity was lower due to pasture with lower nutritional value and lower gains, as well as reduced pasture support capacity, preventing higher stocking rates. Beef productivity per t of feed input (pasture, purchased forages, and concentrated feed) was greater for grain supplementation compared to other systems (Table 4). 

If we consider this meat production for human consumption, we can quantify the importance of intensifying production in the same area. The global projection for per capita consumption of meat in 2028 is 35.1 kg/person/year [38]. Considering the increase in meat production in the strategies we evaluated, GS would produce 92 kg of meat CW/ha more than the extensive system, so it is possible to feed about 2.63 more people per hectare. For the PS and PF systems, meat production was 82 kg/ha more than extensive, corresponding to 2.35 more people fed per hectare compared to extensive grazing. Human edible feed conversion efficiency (heFCE) was below one and thus less efficient for the GS system for both protein (0.28 to 0.30) and energy (0.16) content. Pasture re-seeding, pasture fertilization, and extensive grazing had heFCE values greater than one for protein but less than one for energy. GS’s heFCE was 87% to 94% lower for protein and 60% to 79% lower for energy compared to other systems (Table 5).

### 3.4. Profitability of Simulated Beef Production Systems

For Cerrado and Amazon beef simulations, the cost of production per hectare (ha) was lowest for extensive, followed by grain supplementation, pasture re-seeding, and pasture fertilization. However, for Cerrado beef simulations since revenues were lower, the extensive system had a lower net return (US$22.86 ha/year) compared to the three sustainable agricultural intensification (SAI) strategies, due to lower animal stocking rate and lower beef productivity. Net returns increased with PS (US$61.08 ha/year) and PF (US$65.69 ha/year), with the highest net return for GS (US$92.26 ha/year). Amazon beef simulations were similar with net returns per ha per year lowest for extensive (US$24.28 ha/year), followed by PS (US$64.26 ha/year), PF (US$66.75 ha/year), and then highest for GS (US$90.01 ha/year). While all three SAI strategies have similar revenues, GS was more profitable compared to PS and PF due to lower crop production and animal purchases, despite higher cost of purchased feed (Table 6). The relative profitability of all systems did not change when comparing annual profits growing at a compound interest rate of 6% for each system.

### 3.5. Environmental Impacts

For all systems evaluated in both biomes (Table 7), the main sources of greenhouse gas (GHG) emissions were from cattle. Extensive grazing presented the largest carbon footprint in both biomes. In the Cerrado biome, the carbon footprint (CF in kgCO_2_ eq/kg body weight or kgCO_2_ eq/kg carcass weight) for grain supplementation was 84% of the CF for the extensive baseline scenario. GS had the lowest CF due to higher feed efficiency resulting in reduced emissions per kg of beef body weight (BW) and per kg of beef carcass weight. Pasture re-seeding and pasture fertilization had higher CF than GS due to accumulated emissions to produce and apply fertilizers and other inputs in addition to greater methane emissions from cattle (Supplementary Materials, Tables S3 and S4). Amazon biome CF results were similar with the CF of GS also 84% of extensive. PS and PF also had greater CF’s than GS. Emissions per hectare for sustainable agricultural intensification strategies were more than double than extensive due to doubled stocking density (Table 7).

Water footprint (kg/kg BW or kg/kg CW) for both biomes is greatest for GS. More dietary concentrated feed increased total water consumption by cattle. More water was also needed for grain supplement production. Other systems (PS, PF, and extensive) had water footprints that are only a certain percentage of GS. For the Cerrado biome, this ranged from 87% to 92%, while in the Amazon this was 63% to 88% (Table 7). PS and PF has 5% to 13% less water use for pastures (Supplementary Materials, Table S4). Water use per hectare was greatest for GS followed by extensive and then pasture fertilization and pasture re-seeding (Table 7).

Energy footprint (EF = MJ/kg BW or MJ/kg CW) includes all energy entering the system, directly and indirectly, responsible for beef production. Across both biomes, Cerrado PF had both the highest energy use per area (4240 MJ/ha) and highest EF due to fertilizers applied to pastures providing a greater energy input to the system, which was 9% to 27% greater than the other systems. GS had the lowest EF. Results were similar for the Amazon biome where the EF for PF was 9% to 47% greater than other systems. GS also had the lowest EF of all systems. Energy use per hectare was greatest for PF, PS, and GS followed by extensive (Table 7). 

Nitrogen losses were at least double to almost triple for the three sustainable agricultural intensification systems (~54–74 kg/ha) compared to extensive pasture (~28 kg/ha). Reactive nitrogen footprint for both biomes was lowest for grain supplementation. For the Cerrado biome, GS was 74% to 87% of the reactive nitrogen footprint (RNF) of the other systems. Similarly, for the Amazon biome, GS was 86% to 98% of the RNF of other systems. Nitrogen losses per ha were greatest for PF, then PS, followed by GS and extensive grazing (Table 7).

## 4. Discussion

### 4.1. Sustainable Agricultural Intensification’s Beef Productivity, Feed Conversion Efficiency, and Profitability

To meet the growing demand for food, cattle herds are expected to increase 73% by 2050. Given this increase, there would be no “pioneer frontiers” to convert into agriculture [39]. Brazilian agricultural production could increase without further deforestation and conversion of natural habitat to beef production [40]. Simulated beef weight sold at maturity (kg/ha/year) in the Cerrado and Amazon increased from extensive natural pasture (~124) to pasture re-seeding and fertilization (~280) to grain supplementation (~300). Thus, adopting the sustainable agricultural intensification strategies we evaluated can increase beef production from 126% to 144%.

In extensive systems with pasture degradation, beef production can be as low as 40–50 kg/ha/year with dry season stocking rate less than 0.5 head/ha [41,42]. Our productivity results for the extensive model were double to triple this productivity range since pastures were not at an advanced stage of degradation. For our IFSM simulations, decline in dry season pasture production was compensated by forage purchases via the model’s linear programming (Table 4). IFSM does not allow for changing stocking density (e.g., 1 head/ha for extensive simulations) in the same calendar year. Instead of feeding purchased forage during the dry season, Brazilian ranchers can reduce stocking rate thus distributing cattle over more pasture area (e.g., 0.5 head/ha). 

Our simulated beef production increase of ~127% for pasture re-seeding (PS) was greater than the 107% increase modeled using linear programming for Nelore in nearby Mato Grosso do Sul state [43]. It was also within projected Amazon Nelore production increases of 100% to 251% after 6 to 12 years of pasture restoration in Pará state [44] and close to 130% to 150% measured for Acre state [45]. Our results were consistent with prior research [32], where, compared with the extensive system, pasture systems using intensification strategies had higher costs, but beef production almost doubled with combined intensification techniques including pasture fertilization [32]. Our modeled strategies had higher production costs, but meat production and profitability were also higher than extensive, as system intensification is required to achieve higher slaughter weight and carcass yield which dilutes costs per kg of beef [46].

Optimal pasture fertilization (PF) improves pasture stands insuring complete coverage of soil by forage in its productive stage given adjustments to stocking rates in dry versus wet seasons [41]. For Nelore cross-bred cattle in the Cerrado, forage availability of one-year-old pasture was 197% greater than 20-year-old pasture, allowing for a stocking rate of 3 head/ha [47]. Our results were consistent with this, where PF’s yield and forage supply during the simulation’s first year was ~245% greater in Amazon and Cerrado biomes compared to extensive pasture. 

Grain supplementation (GS) can correct grazing nutritional deficiencies, decrease grazing pressure, and improve cattle performance and reducing time to slaughter [48]. In tropical regions with low rainfall during the dry season, pasture production decreases. Grain supplementation allows animal productivity to remain constant during the dry season, avoiding weight loss during this time. Compared with the extensive system, our GS age at slaughter was reduced by four months for PF and PS and 6 months for GS. GS had the highest yield (~300 kg/ha/year) among modeled systems. Farms in the Amazon that adopted supplementation for Nelore cattle reduced slaughter age by 3.4 months and increased slaughter rates [16]. In the Cerrado, when forage was improved and supplementation provided, Nelore cross-bred cattle growth rate increased while total annual dry matter intake decreased due to better forage quality and digestibility [14]. 

Despite greater beef production, the GS system used more human edible protein and energy from corn grain and soybeans to feed the herd, which are feed sources that compete with human food. Thus, even though GS produced more beef per hectare (Table 4), less total food may be available to humans. GS had human edible feed conversion efficiency (heFCE) below the other beef systems for both protein and energy (Table 5), indicating a net reduction in potential human food driven by greater use of human edible feed to produce animal products. Thus, from a heFCE perspective, beef production that is more grass-based is more favorable than systems like GS feeding more human edible concentrated feed inputs. Our heFCE ratios for protein were comparable to those estimated for the U.S. beef industry [49] with our extensive and improved pasture systems (2.4 to 4.37) less than U.S. beef stockers (5.22) and our GS system (0.28 to 0.32) similar to U.S industrial feedlots (0.34). Despite lower beef production per hectare than GS (Table 4), pasture intensification may provide a net gain in human food supply since it uses less concentrated feeds that can been eaten by humans.

Our simulations’ whole-farm annual profits for pasture improvements (~US$65/ha) and grain supplementation (~US$91/ha) were consistent with other Brazilian studies for Nelore cattle. Whole-farm profits ranged from US$57 to 91/ha for pasture improvements including re-seeding in Acre state in the Amazon [45]. Profits were US$66.72/ha for pasture fertilization in Minas Gerais state in Brazil’s Atlantic Forest biome [50]. Pasture-based grain supplementation (GS) economic analyses have previously been limited to two studies on temperate cattle finished on the productive, diversified temperate pastures in Brazil’s southern Pampas biome. Pasturing on soybean residues increased farm profits to US$124/ha compared to US$ 81/ha for native pasture [51]. For *Bos taurus* in southern Brazil, profits for GS (US$99/ha) was slightly lower compared to native pasture (US$101/ha) [18].

### 4.2. Reducing Carbon Footprint Using Pasture Improvements and Grain Supplementation

For all simulated systems, our results are consistent with a study of Amazon Nelore, where pasture-based beef production system intensification can increase productive efficiency and mitigate certain environmental impacts [52]. The reduced carbon footprint (kgCO_2_ eq/kg BW) for our pasture re-seeding simulations (~6% less) was lower than other studies for Brazilian Nelore cattle [14,43,53] which ranged from 19% to 61% less. However, our simulated pasture improvements had greater indirect emissions from land management and production of external resources compared to extensive due to livestock feed and fertilizer production and use. Emissions from fuels were also higher for pasture re-seeding and fertilization due to machinery use.

For *Brachiaria brizantha*, accumulated carbon footprint (CF) over time was higher for fertilized pasture compared to degraded pasture [42]. In our improved pasture scenarios, GHG emissions per hectare were greater than other systems. As farms increase herd size and produce more pounds of meat per year on the same amount of land, emissions per hectare increase while emissions per kilogram of meat produced tend to decrease. Therefore, increased productivity lowers CF (kg CO_2_ eq/kg BW) for intensified versus extensive systems [16]. 

Our simulated reduction in CF for pasture fertilization was consistent with prior research [16,32]. Pasture fertilization reduces grazing time due to better forage availability. Forage degradation by rumen microbiota is enhanced by lower amounts of cellulose in young plants. Better pasture productivity maintains constant leaf cover and a favorable carbon/nitrogen equilibrium [47,53]. Further lowering CF by increasing percent of pasture area fertilized beyond 10% eventually runs into cost limitations. Profits are negative at roughly -US$45/ha/year (data not shown) in both biomes if 100% of pasture is fertilized annually. 

Our lower carbon footprint (CF) for grain supplementation (GS) was consistent with a study of Nelore in the Amazon where grain supplementation reduced greenhouse gas emissions per kilogram of meat compared to traditional systems [54]. Ruminants fed high-grain diets have lower methane emissions compared to diets with more forage fiber [55]. The 16% reduction in CF for our GS simulations was within the range of 11% to 45% for studies involving Nelore crosses [56] and Nelore pure bred cattle [14].

The sustainable agricultural intensification strategies we modeled lowering Brazil Nelore beef carbon footprint is significant since cattle production makes up 96% of beef sector emissions [57]. While our IFSM simulations focus on such cattle production, they do not model greenhouse gas (GHG) emissions from the deforestation process itself, where burning releases CO_2_ and subsequent extensive pastures have more limited carbon sequestration potential [58]. For example, after annualizing over a 20-year period, carbon footprint of Brazilian beef solely raised on deforested land is 726 kg CO_2_ eq/kg of body weight [59], which is ~40 times greater than the CF for the beef systems we modeled. 

Brazil has recently relied on public policies to reduce deforestation [60]. The Brazilian Forest Code mandates legal forest reserves of 80% in the Amazon and 20% in the Cerrado of property owned in rural areas [61]. The National Climate Change Plan targets reducing deforestation in the Amazon and Cerrado, while the Agricultura de Baixa Emissão de Carbono (ABC) plan encourages low-carbon agricultural emissions [62]. There are also Brazilian government programs for degraded pasture recovery [63]. For other areas of Brazil’s beef value chain, such as product marketing and distribution, system-wide modeling can be essential for determining systems with lower GHG emissions [64].

### 4.3. Carbon Emissions, Water, Energy, and Nitrogen Impacts of Intensification

Reducing Brazilian beef’s carbon footprint is beneficial since Brazil is projected to increase beef production and exports [65] (USDA 2019). While it is important to determine which production systems can best lower GHG emissions per kg of beef produced, our results demonstrate that total GHG emissions per pasture area (kg CO_2_ eq/ha) can more than double on existing pastures if stocking density is doubled from one to two head per hectare. So, while our simulations indicate pasture improvements and grain supplementation can reduce carbon footprint (kg CO_2_ eq/kg of beef body weight) by ~5% to ~16% compared to extensive pasture, this is less than CF reduction of almost 60% required for GHG emissions per hectare for the alternative systems we modeled to equal extensive pasture at half the stocking density. In the long run, reducing total GHG emissions by doubling Brazil’s beef production on the same pasture area may be challenging. However, in the short run, annualized GHG emissions may be lowered by reducing age to slaughter from the Brazilian average of four years [66] to two years. IFSM currently limits age to slaughter at three years.

Reducing both current and future GHG emissions per area and per kg of beef produced in Brazil is critical due the contribution to such emissions from cattle. Enteric fermentation from microbial decomposition of food in the rumen releases methane (CH_4_), which is the largest proportion of total GHG emissions in beef cattle production systems [32]. Beef and dairy cattle are estimated to contribute to 68% of total Brazilian CH_4_ emissions [67] and 56% of total agricultural GHG emissions nationwide [68]. In addition to enteric fermentation from cattle, other GHG sources include manure storage and land application releasing CH_4_ and nitrous oxide (N_2_O), as well as N_2_O and CO_2_ from feed production [58].

Deforestation restrictions, especially in the Amazon biome, increasing agricultural land use and appreciation, and the need to control pasture degradation and greenhouse gas emissions have all contributed to producers reconsidering traditional extensive beef grazing [69]. Our results were consistent with the SimPec model of Mato Grosso state Nelore cattle which showed grain supplementation had more favorable reduction in CF compared to pasture restoration [70]. However, improving degraded pasture and grain supplementation may not be enough to reduce total and per hectare GHG emissions. Although producers have been slow to adopt integrated crop, livestock, forestry systems due to its management complexity [71,72], integrating commercial forestry (e.g., Eucalyptus spp.) can sequester 17,000 kg CO_2_ eq/ha/year [42], which is ~3.5 times the emissions per hectare of the sustainable agricultural intensification systems we evaluated.

Many low input, predominantly non-irrigated livestock systems have little impact on freshwater resources from consumable water use [73]. Due to the higher proportion of concentrate in the diet, the total water consumption per hectare and per kg of beef body weight by animals is higher for GS consistent with prior research [32,74]. Our simulated water use for pasture improvements was higher per kg of beef body weight (BW) compared to extensive, but was lower per hectare in the Cerrado (−13%) and Amazon (−5%) biomes (Supplementary Materials, Table S4). Thus, there appears to be greater responsiveness of reducing water use from improved pasture management for the more water limited Cerrado compared to the less water limited Amazon (Figure 2, Figures S1 and S2).

It is understandable that increased GHG emissions, nitrogen loss, and energy use per ha for our pasture re-seeding/fertilization and grain supplementation models were driven by doubling cattle stocking density. However, even though beef productivity more than doubled, both pasture re-seeding/fertilization and grain supplementation models had greater water footprint (kg/kg cattle BW) and pasture re-seeding/fertilization has greater energy and nitrogen footprints compared to extensive pasture, underscoring the potential environmental impacts that such initial pasture-based intensification entails. If Brazil follows the U.S. beef intensification pathway toward more industrial feedlots, there could be increases in GHG’s, nitrogen losses, and energy use per hectare, water footprint, and also negative external impacts to groundwater and watersheds [75], even though feedlots have been shown to reduce deforestation [76].

## 5. Conclusions

The Integrated Farm System Model software was used to model two cooperating beef farms, one in the Amazon and one in the Cerrado biome in Mato Grosso state, Brazil. The simulated dry matter consumption was consistent with grazing estimates and concentrated feed consumed by beef cattle on the two collaborating farms, demonstrating that the IFSM model can simulate tropical beef systems. When simulating sustainable agricultural intensification strategies, beef production and economic returns were greatest for grain supplementation of cattle followed by pasture re-seeding or pasture fertilization and lowest for extensive pasture. The three sustainable intensification strategies modeled had double the beef cattle stocking density compared to extensive grazing. This increased beef productivity which improved environmental footprints. Grain supplementation, pasture re-seeding, and pasture fertilization all had lower carbon footprint compared to extensive grazing. While grain supplementation had lower energy use and nitrogen loss, these were greater for both pasture management strategies. Water footprint was greater for all three intensification strategies compared to extensive grazing. While grain supplementation had the best beef productivity, economic profitability, and lowest carbon footprint of all simulated systems, intensifying pasture management in Brazil’s Midwest could do better at reducing competition of livestock feed with human food.

## Figures and Tables

**Table 1:** Production system characteristics for the representative farms in the Cerrado and Amazon biomes, Brazil.

Description	Cooperating Cerrado Farm		Cooperating Amazon Farm		
	2016–2017	2017–2018	2015–2016	2016–2017	2017–2018
Farm characteristics					
Production system	Beef stocker	Beef stocker	Beef finish	Beef finish	Beef finish
Pasture type/Crop(s)	*Brachiaria* *brizantha* cv. Marandu	*Brachiaria* *brizantha* cv. Marandu	*Brachiaria* spp./ Rice ^a^	*Brach*. spp./ Rice, Soybean ^a^	*Brach*. spp./ Soybean ^a^
Area, ha					
Grass	930.6	930.6	6147	6747	6747
Crop ^a^	-	-	874	1214	1214
Forest	343.35	343.35	16,596	16,596	16,596
Soil characteristics					
Soil type(s)	P ^b^	P ^b^	DRYL; QN ^b^	DRYL; QN ^b^	DRYL; QN ^b^
% sand, silt, clay	65%, 9%, 26%	65%, 9%, 26%	77%, 6%, 17%	77%, 6%, 17%	77%, 6%, 17%
H_2_O capacity (mm)	260	260	400	400	400
Livestock					
Breed	Nelore (*Bos indicus*)	Nelore (*Bos indicus*)	Nelore (*Bos indicus*)	Nelore (*Bos indicus*)	Nelore (*Bos indicus*)
Production cycle	Calves, replacement	Calves, replacement	Full cycle	Full cycle	Full cycle
Diet	Pasture/ Supplem. ^c^	Pasture/ Supplem. ^c^	Pasture/ Supplem. ^c^	Pasture/ Supplem. ^c^	Pasture/ Supplem. ^c^
Herd, head	1516	2344	15442	11,934	12,170
Stocking rate					
Head/ha	1.63	2.52	2.51	1.77	1.80
kg/ha	441.35	696.14	991.84	749.36	704.26
Annual production Arrobas (30 kg/ha)	-	-	8	8.1	8.1
kg/ha	-	-	240	243	243

^a^ Cooperating farm in Amazon used rice and soybeans as cash crops integrated with pasture areas. ^b^ Predominant soil for Cerrado cooperating farm is plinthosol (P). Predominant soil for Amazon cooperating farm is dystrophic red-yellow latosol (DRYL); part of the farmland has quartzarenic neosol (QN). ^c^ Low-grain supplementation soybean and corn meal, and cottonseed with minerals for finishing cattle and/or stockers.

**Table 2:** Pasture area, beef cattle herd size and stocking rate, fertilization, growth period objectives, cattle feeding and body weight goals for simulations of the extensive beef system, pasture seeding (PS), pasture fertilization (PF), and grain supplementation (GS) in the Cerrado and Amazon biomes, Brazil.

Parameters	Simulated Scenarios in Cerrado and Amazon Biomes			
	Extensive	PS	PF	GS
Land area (ha)				
Pasture	1200	1200	1200	1200
Cattle (head)	1200	2400	2400	2400
Stocking rate (head/ha)	1.0	2.0	2.0	2.0
Fertilizer application rate				
Nitrogen (kg N/ha)	0	50	100	0
Phosphate (kg P_2_O_5_/ha)	0	20	50	0
Potash (kg K_2_O/ha)	0	20	50	0
Lime (metric ton/ha/year)	0	1.5	1.5	0
Pasture				
Re-seeding	No	Yes	No	No
Years between re-seeding	n/a	10	n/a	n/a
Percent (%) fertilized annually	0	10	10	0
Growing period goals (months)				
Age of weaning	8	7	7	7
Stocker period	11	10	10	10
Backgrounding period	6	0	0	0
Finishing period	11	7	7	5
Supplementation	No	No	No	Yes
Forage to grain ratio	High	High	High	High
Finish shrunk body weight goals (kg)				
Initial	360	360	360	360
Final	430	430	430	430

**Table 3:** Actual versus Integrated Farm System Model (IFSM) simulated annual production and purchases for cooperating beef farms in Cerrado and Amazon biomes.

Beef System Output	Cooperating Cerrado Farm				Cooperating Amazon Farm					
	2016–2017		2017–2018		2015–2016		2016–2017		2017–2018	
	Actual	Simulated	Actual	Simulated	Actual	Simulated	Actual	Simulated	Actual	Simulated
Grazed pasture (t DM/ha)	-	3.03	-	4.89	-	3.66	-	2.97	-	1.79
Calculated ^a^ forage DMI required (t DM/ha)										
Minimum at 1.8%	2.99	-	4.64	-	-	-	-	-	-	-
Maximum at 2.5%	4.15	-	6.44	-	3.94 ^a^	-	2.89 ^a^	-	2.37 ^a^	-
Production (t DM/ha)										
Soybean	-	-	-	-	-	-	2.41	2.62	2.08	2.15
Purchases (t DM/ha)										
Forage	-	0.90	-	1.68	-	0.57	0.31	0.34	0.47	0.30
Corn grain	0.21	0.18	0.34	0.22	0.48	0.52	0.30	0.16	0.35	0.19
Cottonseed	-	-	-	-	0.03	0.04	-	-	-	-
Soybean, 44% protein	0.19	0.24	0.30	0.29	0.14	0.18	0.10	0.14	0.08	0.24
Minerals	0.25	0.18	0.41	0.03	0.02	0.02	0.02	0.02	0.01	0.01
Grazed pasture (t DM/head)	-	2.19	-	1.43	-	5.60	-	6.11	-	3.61
Calculated ^a^ forage DMI required (t DM/head)										
Minimum at 1.8%	1.83	-	1.97	-	-	-	-	-	-	-
Maximum at 2.5%	2.55	-	2.74	-	6.03 ^a^	-	5.95 ^a^	-	4.78 ^a^	-
Purchases (t DM/head)										
Forage	-	0.55	-	0.66	-	0.87	0.64	0.70	0.95	0.61
Corn grain	0.13	0.11	0.14	0.86	0.73	0.80	0.62	0.33	0.71	0.38
Soybean, 44% protein	0.11	0.14	0.12	0.12	0.21	0.28	0.21	0.29	0.16	0.48
Minerals	0.16	0.11	0.16	0.01	0.03	0.03	0.04	0.04	0.02	0.02
Beef production (kg BW/ha)	-	-	-	-	231	267	243	236	289	248

^a^ For cooperating farm in Cerrado biome, calculated from National Research Council (NRC) dry matter intake (DMI) requirements for beef cattle at daily DMI per cattle body weight of 1.8% (minimum) and 2.5% (maximum). For cooperating farm in Amazon biome, calculated from NRC dry matter intake (DMI) requirements for beef cattle at daily DMI/cattle body weight of 2.5%.

**Table 4:** Simulated pasture and feed consumption in IFSM for the extensive beef system, pasture seeding (PS), pasture fertilization (PF), and grain supplementation (GS) in the Cerrado and Amazon biomes, Brazil.

Output of Simulated Beef Systems	Cerrado Simulations				Amazon Simulations			
	Extensive	PS	PF	GS	Extensive	PS	PF	GS
Grazed forage consumed (t DM/ha)	1.61	3.41	3.39	3.11	1.74	3.55	3.53	3.26
Purchased								
Forage (t DM/ha)	0.52	1.25	1.26	1.24	0.30	1.03	1.03	1.02
Grain (t DM/ha)	0.04	0.06	0.06	0.13	0.03	0.05	0.06	0.12
Soybean meal (t DM/ha)	-	-	-	0.11	-	-	-	0.13
Mineral & vitamin mix (t DM/ha)	0.01	0.02	0.02	0.02	0.01	0.02	0.02	0.02
Grazed forage & purchased feed input (t DM/ha) ^a^	12.17	34.72	34.71	34.59	12.07	34.63	34.62	34.53
Grazed forage consumed (t DM/head)	1.61	1.71	1.70	1.56	1.74	1.77	1.77	1.63
Purchased								
Forage (t DM/head)	0.52	0.63	0.63	0.62	0.30	0.52	0.52	0.51
Grain (t DM/head)	0.04	0.03	0.03	0.07	0.03	0.02	0.03	0.06
Soybean meal (t DM/head)	-	-	-	0.06	-	-	-	0.06
Mineral and vitamin mix (t DM/head)	0.01	0.01	0.01	0.01	0.01	0.01	0.01	0.01
Grazed forage & purchased feed input (t DM/head) ^a^	12.17	32.37	32.36	32.31	12.07	32.31	32.32	32.26
Net animal weight sold (kg BW/ha)	123	281	281	300	124	280	280	301
(kg CW/ha)	64	147	147	157	65	146	146	157
Net animal weight sold per feed input (kg BW/t DM)	56.7	59.5	59.7	65.4	59.9	60.5	60.6	66.5
(kg CW/t DM)	29.6	31.1	31.1	34.1	31.3	31.6	31.6	34.7

^a^ Does not include mineral and vitamin mix.

**Table 5:** Human edible feed conversion efficiency calculations for the extensive beef system, pasture seeding (PS), pasture fertilization (PF), and grain supplementation (GS) in the Cerrado and Amazon biomes, Brazil.

Calculations of Simulated Beef Systems	Cerrado Simulations				Amazon Simulations			
	Extensive	PS	PF	GS	Extensive	PS	PF	GS
Human edible feed conversion efficiency (heFCE)								
Protein								
Saleable meat (kg/ha)	48	109	109	116	48	108	108	116
Beef protein sold (kg/ha)	9.0	20.6	20.6	22.0	9.1	20.6	20.6	22.1
Grain (kg DM/ha)	40	60	60	130	30	50	60	120
Grain protein (kg/ha)	3.8	5.6	5.6	12.2	2.8	4.7	5.6	11.3
Soybean meal (kg DM/ha)	0	0	0	110	0	0	0	130
Soybean meal protein (kg/ha)	0	0	0	57.0	0	0	0	67.3
Total Feed Protein (kg protein/ha)	3.8	5.6	5.6	69.2	2.8	4.7	5.6	78.6
heFCE = Beef protein/Human edible feed protein	2.40	3.66	3.66	0.32	3.23	4.37	3.64	0.28
Energy								
Saleable meat (kg/ha)	48	109	109	116	48	109	109	116
Beef energy sold (MJ/ha)	308	703	703	751	310	701	701	753
Grain (kg DM/ha)	40	60	60	130	30	50	60	120
Grain energy (MJ/ha)	748	1122	1122	2431	561	935	1122	2244
Soybean meal (kg DM/ha)	0	0	0	110	0	0	0	130
Soybean meal energy (MJ/ha)	0	0	0	2167	0	0	0	2561
Total Feed Energy (MJ/ha)	748	1122	1122	4598	561	935	1122	4805
heFCE = Beef energy/Human edible feed energy	0.41	0.63	0.63	0.16	0.55	0.75	0.62	0.16

**Table 6:** Revenues, costs, and returns simulated in IFSM for the extensive beef system, pasture seeding (PS), pasture fertilization (PF), and grain supplementation (GS) in the Cerrado and Amazon biomes, Brazil.

Output of Simulated Beef Systems	Simulated Scenarios in Cerrado				Simulated Scenarios in Amazon			
	Extensive	PS	PF	GS	Extensive	PS	PF	GS
Farm revenues (US$/y)								
From animal sales	208,846	438,767	438,467	436,917	209,048	439,520	439,520	436,790
Farm revenues (US$/ha/y)	174.04	365.64	365.39	364.10	174.21	366.27	366.27	363.99
Production costs (US$/ha/y)								
Equipment	33.14	47.52	34.51	33.14	33.14	47.52	34.51	33.14
Facilities	0	1.29	1.29	2.61	0	1.29	1.29	2.61
Energy	5.53	10.71	9.54	7.77	5.53	10.71	9.54	7.77
Labor	12.28	29.33	29.21	20.00	11.37	27.50	27.31	19.09
Seed, fertilizer, chemicals	0	31.30	42.86	10.56	0	31.30	42.86	10.56
Purchased feeds	7.15	14.15	14.65	51.58	6.63	13.62	13.94	54.63
Animal purchase & livestock expense	62.19	136.66	136.66	115.11	62.37	139.09	139.09	115.11
Property tax	30.89	30.98	30.98	31.07	30.89	30.98	30.98	31.07
Total cost (US$/ha/y)	151.18	301.94	299.70	271.84	149.93	302.01	299.52	273.98
Net return (US$/ha/y)	22.86	63.70	65.69	92.26	24.28	64.26	66.75	90.01

**Table 7:** Annual average emissions of total greenhouse gases (GHG) and natural resource use per hectare simulated in IFSM for the extensive beef system, pasture seeding (PS), pasture fertilization (PF), and grain supplementation (GS) in the Cerrado and Amazon biomes, Brazil.

Impacts of Simulated Beef Systems	Cerrado Simulations				Amazon Simulations			
	Extensive	PS	PF	GS	Extensive	PS	PF	GS
Greenhouse Gas (GHG) Emissions								
Total GHG emissions (kg CO_2_ eq/ha)	2376	5104	5153	4876	2339	4909	4942	4793
Carbon footprint (kg CO_2_ eq/kg BW)	19.26	18.15	18.32	16.25	18.91	17.50	17.62	15.93
Carbon footprint (kg CO_2_ eq/kg CW)	36.90	34.77	35.10	31.13	36.23	33.52	33.75	30.52
Water Use								
Feed, drinking, & production (Mg/ha)	8368	7960	7904	9288	13,071	12,916	12,969	13,844
Water footprint w/o rain (kg/kg BW)	3340	3403	3450	3769	2049	2825	2852	3256
Water footprint w/o rain (kg/kg CW)	6398	6519	6609	7220	3925	5412	5464	6238
Energy Use								
Feed & resource inputs (MJ/ha)	1496	3888	4240	3567	194	3428	3764	3139
Energy footprint (MJ/kg BW)	13.26	14.68	15.94	12.59	9.60	13.08	14.27	11.14
Energy footprint (MJ/kg CW)	25.40	28.12	30.54	24.12	18.39	25.06	27.34	21.34
Reactive Nitrogen (N) Loss								
Total nitrogen losses (kg/ha)	27.61	70.17	74.16	58.33	27.52	54.18	57.36	57.20
Reactive nitrogen footprint (g/kg BW)	223.0	248.8	263.0	194.6	221.9	193.6	204.9	189.8
Reactive N footprint (g/kg CW)	427.2	476.6	503.8	372.8	425.1	370.9	392.5	363.6

## Supplementary Materials

The following are available online at https://www.mdpi.com/2076-2615/10/8/1386/s1, Table S1: General costs, livestock expenses/year (US$/head), commodity prices (US$/metric ton of dry matter), cattle prices, useful life of capital (years), salvage value (% of initial value), interest rate (%) and fertilizer prices (US$/kg) for simulated systems in the Amazon and Cerrado biomes, Brazil, Table S2: Soil characteristics of the simulated systems for the Cerrado and Amazonia biomes, Brazil, Table S3: Annual average emissions of greenhouse gases (GHG) and natural resource use per farm simulated in IFSM for the extensive beef system, pasture seeding (PS), pasture fertilization (PF), and grain supplementation (GS) in the Cerrado and Amazon biomes, Brazil, Table S4: Annual average emissions of greenhouse gases (GHG) and natural resource use per hectare simulated in IFSM for the extensive beef system, pasture seeding (PS), pasture fertilization (PF), and grain supplementation (GS) in the Cerrado and Amazon biomes, Brazil, Figure S1: Precipitation at cooperating farm and Cuiabá weather stations (2015–2018) in the Cerrado biome, Figure S2: Precipitation at the cooperating farm weather station (2015–18) in the Amazon biome.
