# Supplementary material for: Improve Pasture or Feed Grain? Greenhouse Gas Emissions, Profitability, and Resource Use for Nelore Beef Cattle in Brazil’s Cerrado and Amazon Biomes
Source: Animals (Basel). 2020 Aug 10;10(8):1386. doi: 10.3390/ani10081386 (PMC7459503; doi:10.3390/ani10081386)
Supplement: Supplementary file 1 [file animals-10-01386-s001.pdf]

Article

Improve pasture or feed grain?: Greenhouse gas emissions, profitability, and resource use for Nelore beef cattle in Brazil's Cerrado and Amazon biomes

Luana Molossi [1], Aaron Kinyu Hoshide [2], Lorena Machado Pedrosa [1], André Soares de Oliveira [3] and Daniel Carneiro de Abreu [1,*]

[1] AgriSciences, Universidade Federal de Mato Grosso, Sinop, MT 78555-267, Brazil; luana_molossi@hotmail.com (L.M.) pedrosa@agronoma.eng.br (L.M.P.); abreu@ufmt.br (D.C.A.)
[2] Faculty Associate, School of Economics, The University of Maine, Orono, ME 04469 USA; aaron.hoshide@maine.edu
[3] Dairy Cattle Research Lab, Universidade Federal de Mato Grosso, Sinop, MT 78555-267, Brazil; andresoli@uol.com.br
* Correspondence: abreu@ufmt.br; Tel.: +55-66-3515-8574

Received: 5/23/20

Supplemental Materials

Table S1. General costs, livestock expenses/year (US$/head), commodity prices (US$/metric ton of dry matter), cattle prices, useful life of capital (years), salvage value (% of initial value), interest rate (%) and fertilizer prices (US$/kg) for simulated systems in the Amazon and Cerrado biomes, Brazil.

Item	Cerrado and Amazon
General	
Diesel fuel (US$/liter)	0.86
Electricity (US$/liter)	0.11
Labor wage (US$/hour)	3.50
Veterinary and medicine (US$/head)	3.14
Animal hauling ($/head)	6.16
Miscellaneous ($/head)	2.25
Buying prices (US$/metric ton of dry matter)	
Soybean meal 44%	292.55
Corn grain	70.92
Minerals	431.83
Cattle prices	
Finished cattle ($/kg)	1.22
Cull cow (US$/kg of live weight)	1.14
Bred heifer (US$/animal)	233.00
Feeder cattle (US$/kg of live weight)	1.35
Salvage value (%)	
Machinery	10.0
Structures	2.5
Interest rate (% per year)	
Medium term	6.0
Long term	6.0
Fertilizer prices	
Nitrogen (US$/kg N)	1.04
Phosphate (US$/kg P2O5)	1.31
Potash (US$/kg K2O)	0.96
Lime (US$/t)	28.06

Table S2. Soil characteristics of the simulated systems for the Cerrado and Amazon biomes, Brazil.

Soil attributes	Cerrado	Amazon
Soil type	Shallow loam	Deep sandy loam
Available water holding capacity (mm)	260	400
Fraction of available water when stress begins	0.5	0.5
Bare soil albedo	0.13	0.13
Soil evaporation coefficient (mm)	6	6
Moist bulk density of soil (g/cm3)	1.5	1.5
Organic carbon concentration (%)	0.5	1.37
Silt content (%)	9	6
Clay content (%)	26	17
Sand content (%)	65	77
Runoff curve number w/ row crops	73	73
Whole profile drainage rate coefficient	0.48	0.48
pH	6	4.9
Exchangeable acidity	3.5	3.5

Table S3. Annual average emissions of greenhouse gases (GHG) and natural resource use per farm simulated in IFSM for the extensive beef system, pasture seeding (PS), pasture fertilization (PF), and grain supplementation (GS) in the Cerrado and Amazon biomes, Brazil.

**Impacts of simulated beef systems**	**------- Simulated Scenarios in Cerrado -------**				**-------- Simulated Scenarios in Amazon --------**			
	**Extensive**	**PS**	**PF**	**GS**	**Extensive**	**PS**	**PF**	**GS**
Greenhouse Gas Emissions (kg CO_2 eq/farm)								
Animal emissions	2,095,160	4,448,213	4,444,428	4,330,238	2,087,089	4,441,150	4,437,194	4,332,194
Manure emissions	19,606	43,295	43,303	42,291	18,053	41,765	41,708	40,893
Direct and indirect land emissions	511,481	1,016,972	1,057,072	919,331	544,182	859,038	884,321	883,582
Anthropogenic carbon dioxide emission	4,463	84,181	80,206	4,463	4,463	84,181	80,206	4,463
Production of resource inputs	221,014	533,372	557,918	554,432	153,003	463,842	485,978	491,297
**Total**	**2,851,724**	**6,126,033**	**6,182,927**	**5,850,755**	**2,806,790**	**5,889,976**	**5,929,407**	**5,752,429**
Water Use (Mg/farm)								
Feed production, rainfall	9,545,749	8,305,312	8,322,549	9,786,869	15,380,154	14,548,816	14,603,959	15,437,069
Drinking	12,230	22,349	22,514	24,526	11,183	20,816	20,952	23,185
Production of purchased feed and inputs	483,037	1,224,028	1,140,287	1,334,463	293,491	929,648	938,812	1,153,025
**Total**	**10,041,016**	**9,551,689**	**9,485,350**	**11,145,858**	**15,684,828**	**15,499,280**	**15,563,723**	**16,613,279**
Energy Use (MJ/farm)								
Feed production	62,319	179,584	124,637	62,319	62,319	180,137	124,637	62,319
Production of resource inputs	1,733,015	4,485,869	4,962,873	4,217,912	170,610	3,934,078	4,392,008	3,704,807
**Total**	**1,795,334**	**4,665,453**	**5,087,510**	**4,280,231**	**232,929**	**4,114,215**	**4,516,645**	**3,767,126**
Reactive Nitrogen Loss (kg/farm)								
Ammonia emission	6,952	15,222	15,238	16,887	4,680	11,654	11,634	13,342
Nitrate leaching and runoff	21,812	61,066	65,607	44,915	20,083	41,195	44,816	42,219
De-nitrification emissions	3,587	6,125	6,321	5,791	7,747	10,668	10,852	10,935
Fuel combustion emissions	13	36	25	13	13	37	25	13
Production of resource inputs	771	1,755	1,801	2,384	491	1,462	1,500	2,135
**Total**	**33,135**	**84,204**	**88,992**	**69,990**	**33,014**	**65,016**	**68,827**	**68,644**

Table S4. Annual average emissions of greenhouse gases (GHG) and natural resource use per hectare simulated in IFSM for the extensive beef system, pasture seeding (PS), pasture fertilization (PF), and grain supplementation (GS) in the Cerrado and Amazon biomes, Brazil.

**Impacts of simulated beef systems**	**----- Simulated Scenarios in Cerrado -----**				**------ Simulated Scenarios in Amazon ------**			
	**Extensive**	**PS**	**PF**	**GS**	**Extensive**	**PS**	**PF**	**GS**
Greenhouse Gas Emissions (kg CO_2 eq/ha)								
Animal emissions	1,746	3,707	3,704	3,609	1,739	3,701	3,698	3,610
Manure emissions	16	36	36	35	15	35	35	34
Direct and indirect land emissions	426	847	881	766	453	716	737	736
Anthropogenic carbon dioxide emission	4	70	67	4	4	70	67	4
Production of resource inputs	184	444	465	462	128	387	405	409
**Total**	**2,376**	**5,104**	**5,153**	**4,876**	**2,339**	**4,909**	**4,942**	**4,793**
Water Use (Mg/ha)								
Feed production, rainfall	7,955	6,921	6,935	8,156	12,817	12,124	12,170	12,864
Drinking	10	19	19	20	9	17	17	19
Production of purchased feed and inputs	403	1,020	950	1,112	245	775	782	961
**Total**	**8,368**	**7,960**	**7,904**	**9,288**	**13,071**	**12,916**	**12,969**	**13,844**
Energy Use (MJ/ha)								
Feed production	52	150	104	52	52	150	104	52
Production of resource inputs	1,444	3,738	4,136	3,515	142	3,278	3,660	3,087
**Total**	**1,496**	**3,888**	**4,240**	**3,567**	**194**	**3,428**	**3,764**	**3,139**
Reactive Nitrogen Loss (kg/ha)								
Ammonia emission	5.79	12.69	12.70	14.07	3.90	9.71	9.70	11.12
Nitrate leaching and runoff	18.18	50.89	54.67	37.43	16.74	34.33	37.35	35.18
De-nitrification emissions	2.99	5.10	5.27	4.83	6.46	8.89	9.04	9.11
Fuel combustion emissions	0.011	0.030	0.021	0.011	0.011	0.031	0.021	0.011
Production of resource inputs	0.64	1.46	1.50	1.99	0.41	1.22	1.25	1.78
**Total**	**27.61**	**70.17**	**74.16**	**58.33**	**27.52**	**54.18**	**57.36**	**57.20**

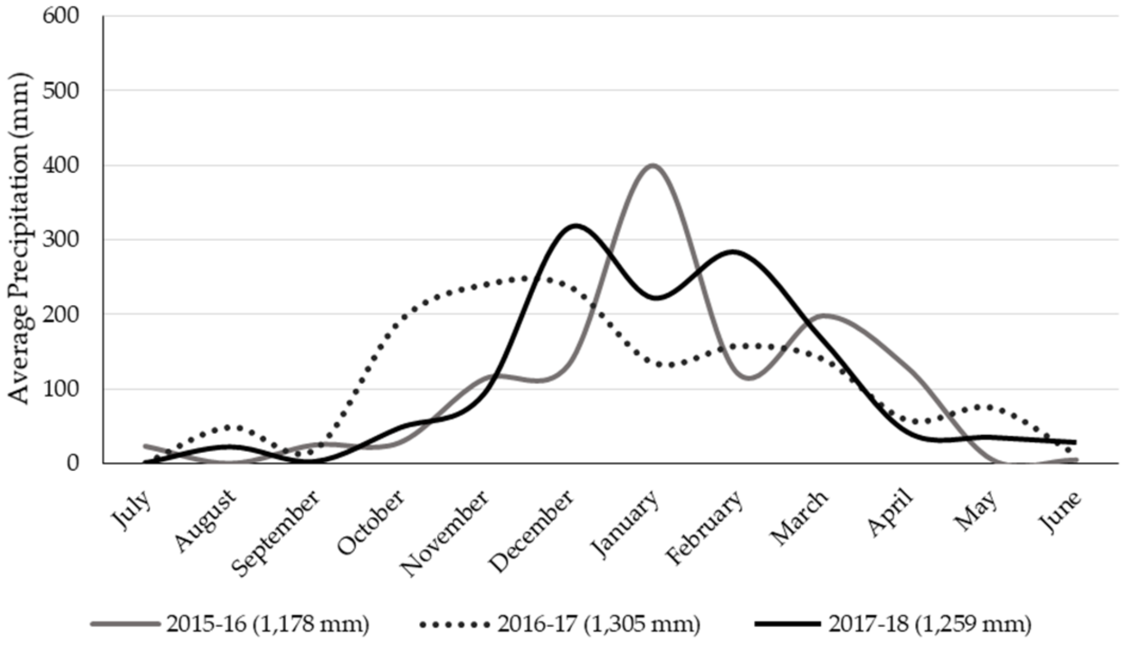

27
28 **Figure S1.** Precipitation at cooperating farm and Cuiabá weather stations (2015-2018) in the Cerrado biome.
29

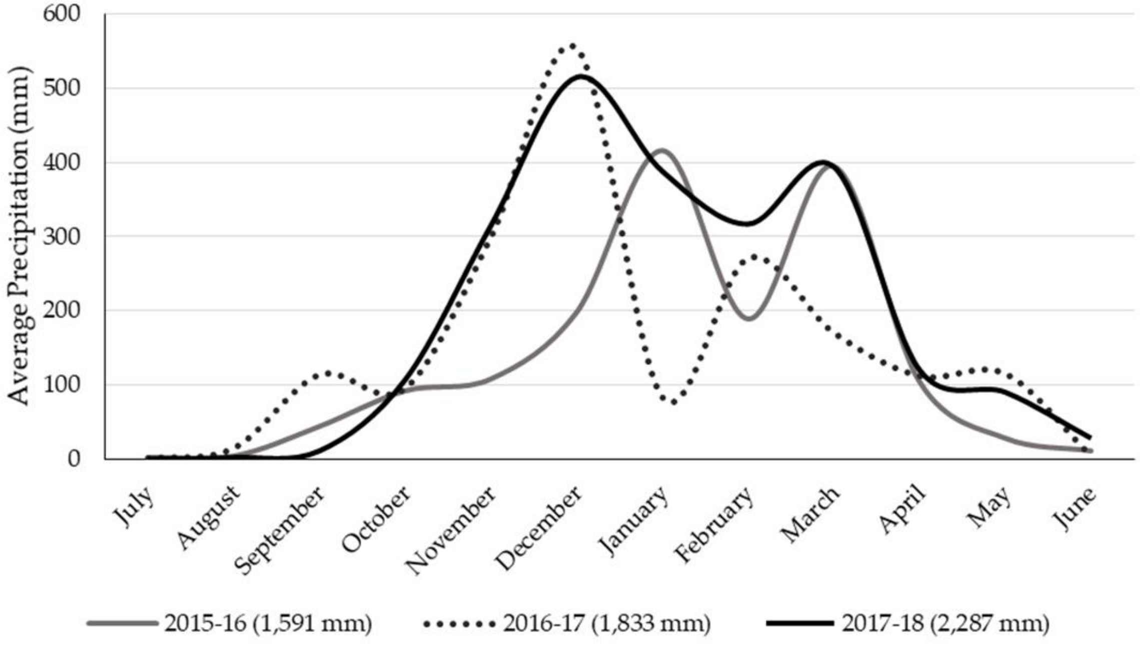

Figure S2. Precipitation at the cooperating farm weather station (2015-2018) in the Amazon biome.

© 2020 by the authors. Submitted for possible open access publication under the terms and conditions of the Creative Commons Attribution (CC BY) license (http://creativecommons.org/licenses/by/4.0/).
